# Implementation of Health Promoting Hospital Standards in Cardiology Hospitals: No Time to Lose Anymore!

**DOI:** 10.34172/aim.2022.66

**Published:** 2022-06-01

**Authors:** Mohammad Mohammadi, Akbar Shafiee

**Affiliations:** ^1^Tehran Heart Center, Cardiovascular Diseases Research Institute, Tehran University of Medical Sciences, Tehran, Iran

## Importance of Cardiovascular Disease

 Cardiovascular diseases are the leading cause of mortality worldwide and have a considerable burden on the healthcare system in every country.^1^ This is also true for Iran, as a developing country, and cardiovascular diseases have been the leading cause of death and disability-adjusted life year (DALY) in Iran for the past decades.^2^ Meanwhile, the burgeoning elderly population in Iran is an alarm for a rapid increase in cardiovascular diseases and thereby their related mortalities in the next few years.^3^ Several health promotion strategies were introduced in Iran to prevent and control cardiovascular risk factors and treat cardiovascular diseases, and one of the main outputs was the establishment of cardiology hospitals.^2^ However, treatment is the routine care provided in these hospitals and other health care centers, which does not seem to lessen the burden of disease over time. Therefore, improvement in the management of these hospitals and extending their mission to consider primary prevention and health promotion as an effective way to decrease the burden of cardiovascular diseases are essential. One of the possible and practical approaches is turning a hospital into a health-promoting hospital (HPH).

## Definition HPH

 The World Health Organization (WHO) launched the Network of Health Promoting Hospitals to accentuate health promotion, disease prevention, and rehabilitation services in medical treatment settings in 1988. The main idea was to commit hospital managers to integrate health promotion into the daily activities of their health care centers. This program aims to redesign the hospital environment and services to empower patients, relatives, and health workers to improve their health-related physical, mental and social well-being. Although some of these activities have been the fundamental works of hospitals’ function throughout history, some tasks have been overlooked, and hospitals generally have a treatment-oriented attitude rather than improving the health status of their clients.^4^ Furthermore, the dramatic rise in the number of patients with lifestyle disorders and chronic diseases requires a more systematic preparation and provision of duties and activities.

 Initially, the WHO formulated five standards for developing and evaluating HPH, which was updated to fulfill the goals and eliminate the drawbacks.^5^ The 2020 standards now include five overarching items:

1. Demonstrating organizational commitment for HPH
2. Ensuring access to the service
3. Enhancing people-centered health care and using involvement
4. Creating a healthy workplace and healthy setting
5. Promoting health in the wider society

The International HPH network currently includes 20 national/regional networks and 50 single members, and almost 600 hospitals and health service members from 30 countries have joined this network so far.

## HPH in Iran

 Although we could not clearly track the introduction of the HPH concept in Iran, studies on HPH in Iran date back to almost a decade ago, and the Persian translation and validation of the standards of HPH was published in 2018.^6^ While a substantial number of the early publications were in the Persian language, recent publications that mostly describe the Iranian experience of HPH are published in English.^7^ A thorough overview of these publications indicates that the standards of HPH were not well-achieved in Iran.^7^ Meanwhile, there were improvements in some indicators such as patient and society empowerment. The treatment-oriented attitude of hospitals is a major pitfall for this failure, which is due to the need for instant financial revenue in the Iranian hospitals.^7^

 According to an interesting qualitative study,^8^ the main barriers to health promotion in hospitals in Iran can be categorized into the following areas:

1. Barriers associated with patients and the community
2. Barriers associated with healthcare professionals
3. Barriers associated with the organization
4. External environment barriers

A review of the studies on HPHs in Iran showed that the most important challenge in achieving the five standards of HPH is the hospital policy. However, we believe all the experiences with HPH in Iran are not documented and this makes any evaluation difficult.

## Implementing HPH Program Into Cardiology Hospitals

 Based on the authors’ survey, the first Iranian cardiology hospital joined the HPH network almost 12 years ago. However, there is no published evidence of this experience, and the Iranian HPH network is currently inactive based on our communication with the International HPH network, and the current members in Iran are only single members (i.e., six hospitals based on the HPH network website at www.hphnet.org). A recent publication evaluated the main health promotion standards at the Farshchian Heart Center of Hamadan that joined the HPH network in 2016.^9^ The authors showed that health promotion policies based on the WHO standards were not well-recognized among patients, hospital staff, and leadership and management staff, and therefore further work is required to achieve the planned goals. Due to the prevalence of cardiovascular diseases, controlling modifiable cardiovascular risk factors can significantly reduce the burden of the disease. Therefore, implementing the HPH program in cardiology hospitals could help to improve the patient’s necessity of preventive cardiology clinics and how HPH standards can help.

 Since most of the cardiology hospitals in Iran are affiliated with a medical sciences university, and they train cardiology residents and fellows, implementing an HPH program can also help to improve the knowledge, attitude, and practice of the trainees toward prevention and health promotion. It is evident that they will carry the message to other centers when they graduate from these hospitals, and in the long term, the whole health care system will benefit from their experience.

## Recommendations

 First, and based on the reports from Iran, we highlight the need for re-establishing the national HPH network in Iran to provide a robust system for observing and evaluating the hospitals and providing a consistent level of health promotion policy based on the defined standards. Second, all physicians and hospital managers are encouraged to provide a comprehensive source of evidence for implementing HPH more extensively in Iran by publishing their experience to perform a comprehensive systematic review on this topic later. We also suppose that their knowledge and experience can help to convince the policymakers and legislators to accept the initial costs of implementing HPH standards in order to reduce the burden of cardiovascular diseases in the future. Accordingly, setting HPH membership as a measure or a credit for accreditation is suggested as an effective motivation process.^10^ Finally, collaboration with agencies that design and conduct assessments and health services accreditation can help to support dissemination and encourage the uptake of the HPH standards.

## References

[1] Li Z, Lin L, Wu H, Yan L, Wang H, Yang H (2021). Global, Regional, and National Death, and Disability-Adjusted Life-Years (DALYs) for Cardiovascular Disease in 2017 and Trends and Risk Analysis From 1990 to 2017 Using the Global Burden of Disease Study and Implications for Prevention. Front Public Health.

[2] Sarrafzadegan N, Mohammmadifard N (2019). Cardiovascular disease in Iran in the last 40 years: prevalence, mortality, morbidity, challenges and strategies for cardiovascular prevention. Arch Iran Med.

[3] Shafiee A, van Bodegom D (2012). The necessity for research on the elderly in Iran. J Tehran Heart Cent.

[4] Pezeshki MZ, Alizadeh M, Nikpajouh A, Ebadi A, Nohi S, Soleimanpour M (2019). Evaluation of the health promotion standards in governmental and non-governmental hospitals in East-Azerbaijan. Med J Islam Repub Iran.

[5] Nikpajouh A (2017). Standards of health promoting hospitals: 68 or 40 measurable elements?. Health Promot Perspect.

[6] Nikpajouh A, Shahrbaf MA, Doayie M, Mohseny M, Ebadi A, Alizadeh M (2018). Health promoting hospitals in Iran: Persian translation, cultural adaptation, content and face validation of selfassessment form of the standards of health promoting hospitals affiliated to the World Health Organization. Med J Islam Repub Iran.

[7] Hamidi Y, Hazavehei SMM, Karimi-Shahanjarini A, Seif Rabiei MA, Farhadian M, Alimohamadi S (2019). Health promoting hospitals in Iran: a review of the current status, challenges, and future prospects. Med J Islam Repub Iran.

[8] Afshari A, Mostafavi F, Latifi A, Ahmadi Ghahnaviyeh L, Pirouzi M, Eslami AA (2018). Hospitals reorientation towards health promotion: a qualitative study of barriers to and strategies for implementation of health promotion in hospitals of Isfahan, Iran. J Educ Health Promot.

[9] Seif Rabiei MA, Khanlarzadeh E, Nikpajouh A, Doaee M, Sanaei Z (2020). Evaluating health promotion standards at the Farshchian heart center of Hamadan, Iran. Hosp Pract (1995).

[10] Yousefinezhadi T, Mosadeghrad AM, Arab M, Ramezani M, Akbari Sari A (2017). An analysis of hospital accreditation policy in Iran. Iran J Public Health.

